# Self-prioritization and perceptual matching: The effects of temporal construal

**DOI:** 10.3758/s13421-017-0722-3

**Published:** 2017-06-07

**Authors:** Marius Golubickis, Johanna K. Falben, Arash Sahraie, Aleksandar Visokomogilski, William A. Cunningham, Jie Sui, C. Neil Macrae

**Affiliations:** 1 School of Psychology, University of Aberdeen, King’s College, Aberdeen, AB24 3FX Scotland; 2 Department of Psychology, University of Toronto, Toronto, Canada; 3 Department of Psychology, University of Bath, Bath, England UK

**Keywords:** Social cognition, Self, Temporal construal, Drift diffusion model

## Abstract

Recent research has revealed that self-referential processing enhances perceptual judgments — the so-called self-prioritization effect. The extent and origin of this effect remains unknown, however. Noting the multifaceted nature of the self, here we hypothesized that temporal influences on self-construal (i.e., past/future-self continuity) may serve as an important determinant of stimulus prioritization. Specifically, as representations of the self increase in abstraction as a function of temporal distance (i.e., distance from now), self-prioritization may only emerge when stimuli are associated with the current self. The results of three experiments supported this prediction. Self-relevance only enhanced performance in a standard perceptual-matching task when stimuli (i.e., geometric shapes) were connected with the current self; representations of the self in the future (Expts. 1 & 2) and past (Expt. 3) failed to facilitate decision making. To identify the processes underlying task performance, data were interrogated using a hierarchical drift diffusion model (HDDM) approach. Results of these analyses revealed that self-prioritization was underpinned by a stimulus bias (i.e., rate of information uptake). Collectively, these findings elucidate when and how self-relevance influences decisional processing.

As far as indispensable psychological constructs go, the self occupies a position near the top of the list (Baars, 1988). Pervading core aspects of daily life, it guides cognition, shapes behavioral elicitation, and provides coherence and continuity to the ebb and flow of subjective experience (James, 1890; Neisser, 1988). As Kihlstrom and Klein (1994) observed, “self is the unquestionable, elementary, universal fact of mental life, and the fundamental unit of analysis for a science of mental life” (p. 155). It is little wonder, therefore, that the self is a topic that has intrigued scholars for centuries and continues to engender interest from diverse sections of the academic community (e.g., Baumeister, 1998; Boyer, Robbins, & Jack, 2005; Conway & Pleydell-Pearce, 2000; Gallagher, 2000; Gillihan & Farah, 2005; Heatherton, 2011; Heatherton, Macrae, & Kelley, 2004; James, 1890; Klein, Rozendal, & Cosmides, 2002; H. Markus & Wurf, 1987). For the most part, attention has focused on two basic issues: elucidating how the self impacts cognition and decision making and identifying the neuroanatomical structures that support these activities (Blakemore & Robbins, 2012; Conway, 2005; Conway & Pleydell-Pearce, 2000; Heatherton, 2011; Heatherton et al., 2004; Mezulis, Abramson, Hyde, & Hankin, 2004; Sali, Anderson, & Courtney, 2016; Sheppard, Malone, & Sweeny, 2008). Motivating these complementary lines of inquiry is the assumption that self-relevance (i.e., items associated with the self) exerts a potent influence on stimulus processing (Baumeister, 1998; Conway & Pleydell-Pearce, 2000; Heatherton, 2011).

Examination of the available evidence attests to the effects of self-relevance on a range of cognitive operations, but most notably memory function (Conway, 2005; Conway & Pleydell-Pearce, 2000; Heatherton et al., 2004; Symons & Johnson, 1997). When it comes to recollecting the past, self-referential thinking affords information a reliable benefit in recognition and recall (e.g., Kelley et al., 2002; Macrae, Moran, Heatherton, Banfield, & Kelley, 2004; Maki & McCaul, 1985; Rogers, Kuiper, & Kirker, 1977; Symons & Johnson, 1997). For example, following a task in which participants are required to rate the extent to which personality characteristics are descriptive of both the self and a familiar other (e.g., celebrity, best friend, parent), items encoded in the context of the self are advantaged in memory (i.e., self > other; see Symons & Johnson, 1997). Aside from self-descriptive personality traits, people are also highly adept at recognizing their own actions (Knoblich & Flach, 2003). Whether the outcomes of interest are walking, kinematic aspects of handwriting, the trajectories of darts, excerpts of classical music, or the sound of hands clapping, actors are better at identifying their own behavioral products than the comparable outputs of other individuals (e.g., Beardsworth & Buckner, 1981; Knoblich & Flach, 2001; Knoblich & Prinz, 2001; Repp, 1987; Repp & Knoblich, 2004). Put simply, self-relevance enhances memory performance, even when prior levels of engagement with a stimulus are minimal (Cloutier & Macrae, 2008).

Pertinent to the current investigation, the effects of self-referencing extend beyond memorial outcomes. As noted by Sui and Humphreys, self-relevance also has the capacity to influence people’s perceptual judgments (Humphreys & Sui, 2015; Sui & Humphreys, 2015). This, of course, is by no means a novel idea given previous demonstrations of facilitated processing when participants encounter self-relevant (vs. irrelevant) stimuli, such as their face or name (e.g., Bargh, 1982; Bargh & Pratto, 1986; Imafuku, Hakuno, Uchida-Ota, Yamamoto, & Minagawa, 2014; Keyes & Brady, 2010; Ma & Han, 2010; Moray, 1959; Sui, Zhu, & Han, 2006; Sui & Han, 2007; Wood & Cowan, 1995). These studies are not without limitation, however. As the critical items differ substantially in familiarity (e.g., own face vs. a stranger’s face), it is unclear the extent to which self-relevance per se modulates perceptual processing. Remedying this shortcoming, Sui, He, and Humphreys (2012) recently furnished evidence for the enhanced processing of self-relevant information in a bespoke associative-learning paradigm. Specifically, after coupling arbitrary geometric shapes with person-labels (e.g., circle = you, triangle = best friend, square = stranger), participants’ perceptual-matching judgments (i.e., do the items go together?) were fastest and most accurate for stimulus pairs associated with the self (vs. friend or stranger)—the so-called self-prioritization effect (Sui et al., 2012; Sui, Liu, Moverach, & Humphreys, 2013). It has been suggested that self-relevance triggers prioritized processing by enhancing the salience of stimuli (Humphreys & Sui, 2015; Sui & Humphreys, 2015; Sui, Liu, et al., 2013).

Notwithstanding numerous demonstrations of self-prioritization during perceptual matching (e.g., Sui et al., 2012; Sui, Liu, et al., 2013; Sui, Rothstein, & Humphreys, 2013; Sui, Sun, Peng, & Humphreys, 2014), two outstanding issues merit consideration and further empirical scrutiny. These pertain to the generality and basis of the reported self-prioritization effect. First, how sensitive is this effect to differences in the operationalization of the self? At least among healthy individuals, the overarching message in the available literature is that stimulus prioritization is an obligatory consequence of self-referential processing. To optimize functioning in complex and challenging environments, attention is automatically tuned to favor self-relevant stimuli (Humphreys & Sui, 2015; Sui & Humphreys, 2015). But is this inevitably the case or, as is perhaps more likely, is stimulus prioritization sensitive to differences in self-construal (Trope & Liberman, 2003, 2010)? Second, what is the basis of the self-prioritization effect? Despite widespread evidence that self-relevant stimuli facilitate perceptual judgments (Humphreys & Sui, 2015; Sui & Humphreys, 2015), the precise mechanism through which this effect arises remains unknown. Accordingly, the goals of the current research were twofold—to explore the extent and source of the self-prioritization effect during perceptual matching (Sui et al., 2012).

## Self-construal and perceptual matching

In exploring the self-prioritization effect, research to date has adopted a rigid operationalization of the self that potentially underestimates the nuanced ways in which it may (or indeed may not) impact task performance (Humphreys & Sui, 2015; Sui & Humphreys, 2015). Rather than comprising a unitary, monolithic entity, the self is a multifaceted, flexible construct shaped by the collective influence of long-term knowledge, situational forces, and temporary processing goals (Conway & Pleydell-Pearce, 2000; McConnell, 2011; Roberts & Donahue, 1994). For example, it is widely acknowledged that the self comprises multiple social identities (with associated beliefs, expectations, and values)—derived from membership in various social groups (Tajfel, 1978)—that influence behavior in a context-specific manner. When a particular subcomponent of the self is active, individuals make sense of the world through the lens of the associated knowledge structure (e.g., McConnell, 2011; Oyserman, 2009). Similarly, and of relevance to the current inquiry, it is well established that thinking and doing are highly susceptible to temporal influences on self-construal (see Trope & Liberman, 2003, 2010). A central tenet of construal-level theory (CLT) is that forthcoming events (e.g., going on vacation) can be represented in either a super- or subordinate manner, what matters is when an event is scheduled to occur (e.g., next month vs. next year). Whereas impending events (e.g., a trip to Hong Kong) are characterized in a concrete, detail-rich manner (e.g., booking a hotel, acquiring local currency, finding one’s passport), distant events comprise abstract, decontextualized representations that convey only the gist or general meaning of an episode (e.g., enjoying a well-earned break). In other words, as events become temporally distant, representations decrease in detail and increase in schematic content (see also Vallacher & Wegner, 1985).

Importantly, characterizations of the self display comparable temporal shifts in complexity (i.e., concrete to abstract), a structural phenomenon that has implications for assessments of self-continuity (i.e., the perceived similarity between one’s current and future self). Specifically, overlap between one’s current and future self fluctuates as a function of temporal distance, such that a person feels greater affinity with her potential self in the near than distant future (e.g., Parfit, 1971, 1987; Pronin & Ross, 2006; Schelling, 1984; Thaler & Shefrin, 1981; Wakslak, Nussbaum, Liberman, & Trope, 2008). Indeed, travel deeply into the future and psychological connectivity between these constructs can be severed altogether, resulting in one’s future self acquiring the status of a stranger (Hershfield, 2011; Mitchell, Schirmer, Ames, & Gilbert, 2011; Parfit, 1971, 1987; Pronin, Olivola, & Kennedy, 2008; Pronin & Ross, 2006; Wakslak et al., 2008). Intriguingly, this self-becomes-other effect has also been reported for events in the not-so-distant future (e.g., next month, next semester; Pronin et al., 2008; Pronin & Ross, 2006). These temporal influences on self-construal may act as an important determinant of the self-prioritization effect (Humphreys & Sui, 2015; Sui & Humphreys, 2015). Such a boundary condition would have important implications for contemporary theoretical accounts of self-prioritization, as it would challenge the viewpoint that self-biases are a mandatory characteristic of perceptual processing (Humphreys & Sui, 2015). Of course, what has also yet to be established is the specific mechanism that underpins the self-prioritization effect during perceptual matching (Sui et al., 2012).

In exploring the origins of decisional bias, prior research has identified two distinct pathways through which top-down knowledge (e.g., self-relevance) can influence task performance (Ashby, 1983; Leite & Ratcliff, 2011; Link & Heath, 1975; Summerfield & de Lange, 2014; van Ravenzwaaij, Mulder, Tuerlinckx, & Wagenmakers, 2012; White & Poldrack, 2014). During decision making, adjustments can be made to how the stimulus under consideration is evaluated (i.e., stimulus bias) or the manner in which a response is prepared (i.e., response bias). That is, biases can be traced to how sensory information is converted into decisional evidence or how that evidence is used to generate a decision (White & Poldrack, 2014). In terms of the self-prioritization effect, the prevailing viewpoint is that self-relevance influences the perceptual operations that underpin decision making (Humphreys & Sui, 2015; Sui & Humphreys, 2015) and thus represents a bias in stimulus processing. To demonstrate such a bias, however, necessitates the decomposition of task performance during perceptual matching to isolate the specific cognitive process that underpins the self-prioritization effect (Sui et al., 2012). Importantly, in the context of binary decision tasks, drift diffusion models provide just such an opportunity (e.g., Ratcliff, 1978; Ratcliff & Rouder, 1998; Ratcliff, Smith, Brown, & McKoon, 2016; Voss, Nagler, & Lerche, 2013; Voss, Rothermund, & Brandtstädter, 2008; Voss, Rothermund, & Voss, 2004; Voss & Voss, 2007; Voss, Voss, & Lerche, 2015; Wagenmakers, 2009).

## Decomposing the self-prioritization effect

**Fig. 1:**
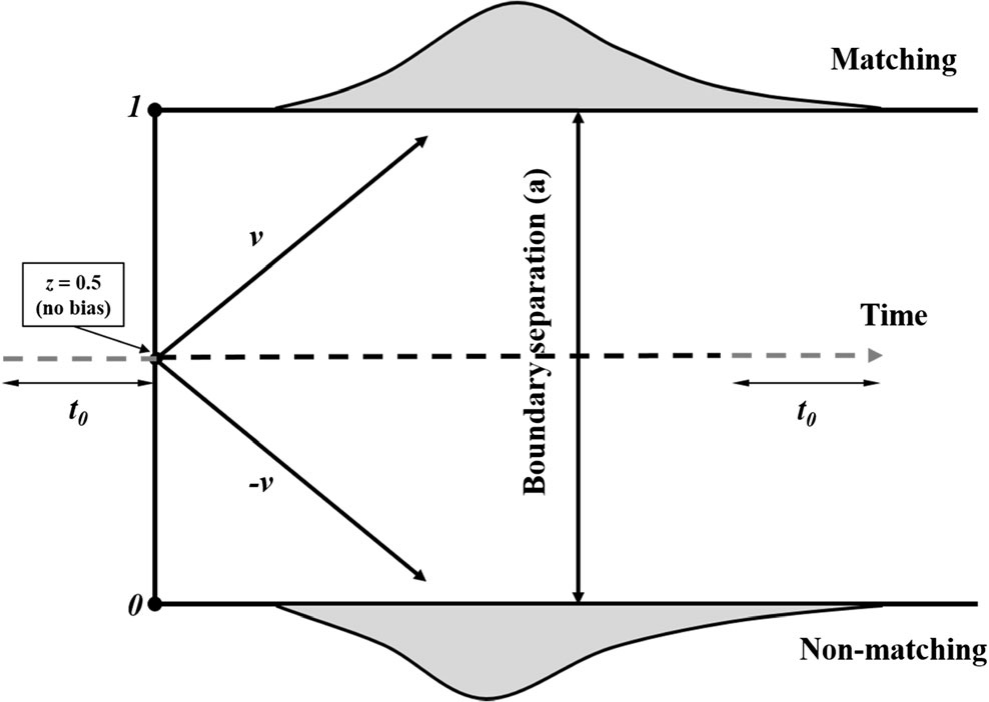
Schematic version of the drift diffusion model, adapted from Voss, Rothermund, et al., (2013, p. 4). An information gathering process begins at starting point *z* and continues with a mean slope *v* until it reaches an upper (*a*) or lower (*0*) threshold. The process durations and outcomes vary from trial to trial because of random noise. Outside the threshold boundaries the decision-time distributions are shown

Drift diffusion models decompose behavioral data (i.e., response times & accuracy) into a set of latent parameters that represent the cognitive operations underlying decisional processing (Voss, Nagler, et al., 2013; Voss et al., 2015). A variant of continuous sampling approaches, these models assume that information is continuously gathered during a decision phase until sufficient evidence is acquired to initiate a response. A schematic depiction of the model is provided in Fig. 1. The model describes evidence accumulation unfolding over time and fits accuracy and response time distributions. The duration of the diffusion process is known as the decision time and the diffusion process itself can be characterized by several important parameters (Voss et al., 2004; Voss, Rothermund, et al., 2013; Voss et al., 2015).^1^

The drift rate (*v*) maps the speed and quality of information acquisition (i.e., larger drift rate = faster information uptake), thus provides a measure of stimulus processing (i.e., stimulus bias) during decision-making (White & Poldrack, 2014). Threshold separation (*a*) estimates the distance between the decisional boundaries, hence indexes how much evidence is sampled before a decision is made (i.e., higher estimates represent thresholds that are most distinct from each other, suggesting greater certainty is required before a response is initiated). The starting point (*z*) defines the position at which evidence accumulation begins. If *z* is not centered between the thresholds, this indicates an a priori bias in favor of the outcome that is closer to the starting point (i.e., response bias). Finally, the duration of all nondecisional processes (i.e., operations occurring pre/post decision making) is given by the additional parameter *t*_*0*,_ and is taken to indicate biases in stimulus encoding and response execution (Voss, Nagler, et al., 2013; Voss et al., 2015).

Drift diffusion modeling is useful in the current context as it has the capacity to isolate the cognitive processes underlying decision making (White & Poldrack, 2014), thereby elucidate the origin of the self-prioritization effect. If, as has been suggested, self-relevance influences the perceptual component of the decisional process (Humphreys & Sui, 2015; Sui & Humphreys, 2015), then self-prioritization should be underpinned by differences in the rate of information uptake (i.e., stimulus bias, *v*) during the standard shape-label matching task (Sui et al., 2012).

## Overview

In three experiments, the goals of the current research were to explore when and how self-relevance impacts the emergence of the self-prioritization effect during perceptual matching (Humphreys & Sui, 2015; Sui & Humphreys, 2015). Noting fundamental temporal influences on self-construal (Hershfield, 2011; Mitchell et al., 2011; Pronin et al., 2008; Pronin & Ross, 2006; Wakslak et al., 2008)—specifically, that representations of the self increase in abstraction as a function of temporal distance—we expected the self-prioritization effect during perceptual matching to be restricted to geometric shapes associated with temporally proximate (vs. distant) representations of the self. We explored this hypothesis in a standard perceptual matching task in which participants associated labels pertaining to the self (e.g., self now, self tomorrow) and a stranger with various geometric shapes (e.g., circle, diamond), then judged (as quickly and accurately as possible) whether subsequent shape-label pairings matched or mismatched the previously learned associations (see Sui et al., 2012). To identify the decisional processes underlying task performance, data were submitted to a HDDM analysis (Wiecki et al., 2013). Of theoretical interest was establishing the extent to which self-relevance influences the rate at which evidence is acquired (i.e., drift rate, *v*) during perceptual matching.^2^ Experiments 1 and 2 explored prospective representations of the self, Experiment Experiment 3 representations of the self in the past.

## Experiment 1

### Participants and design

Sixteen undergraduates (one male, *M*_age_ = 19.69 years, *SD* = 1.89) took part in the research, for which they received £5 (~$6.20).^3^ All participants had normal or corrected-to-normal visual acuity. Informed consent was obtained from participants prior to the commencement of the experiment, and the protocol was reviewed and approved by the Ethics Committee at the School of Psychology, University of Aberdeen. The experiment had a 4 (shape category: self-now vs. self-year vs. self-forty vs. stranger) × 2 (trial type: matching vs. nonmatching) repeated-measures design.

### Stimulus materials and procedure

Participants arrived at the laboratory individually, were greeted by an experimenter, and told they would be performing a perception task. Following Sui et al. (2012), the experiment had two phases. The first phase comprised a learning task in which participants were required to associate geometric shapes (i.e., circle, horizontal rectangle, cross, diamond) with four targets: self-now, self-in-one-year, self-in-forty-years, and a stranger. To bolster the temporal manipulation and trigger construal-based processes, participants engaged in periods of guided imagery (i.e., a simulation for each target) during which they formed target-shape associations. Based on prior research, participants were instructed to close their eyes and imagine the self (now, in-one-year, in-forty-years) or a stranger walking along a quiet beach (Macrae et al., 2015).^4^ After 20 seconds had elapsed, participants were instructed to represent the target of the respective simulation (i.e., self-now, self-one, self-forty, stranger) with a specific geometric shape (i.e., circle, horizontal rectangle, cross, diamond). The shapes were not presented at this stage. The order of the mental simulations and shape-target associations were counterbalanced across the sample.

Next, participants were seated in front of a desktop computer and informed they would be performing a perceptual-matching task. Using two buttons on the keyboard (i.e., N & M), participants had to report whether a series of shape-label pairings (e.g., circle & self-now, cross & stranger, rectangle & self-year, diamond & self-forty) were correct (or incorrect) on the basis of the associations learned previously. Each trial began with the presentation of a central fixation cross for 500 ms, followed by the pairing of a shape (i.e., circle, rectangle, cross, diamond) and label (e.g., self-now, self-one, self-forty, stranger) above and below the fixation cross, respectively, for 100 ms. After each shape-label pairing was presented, the screen turned blank for a variable interval (i.e., 800 ms to 1,100 ms). Participants had to judge the accuracy of the pairings (i.e., whether they matched or mismatched the associations learned earlier) by pressing the corresponding button as quickly as possible within this time frame to encourage immediate responding (Sui et al., 2012). The meaning of the response buttons was counterbalanced across participants. Feedback (i.e., correct or incorrect response) was given on the screen for 500 ms at the end of each trial and participants were also informed of their overall accuracy at the end of each block of trials. Participants initially performed 18 practice trials, followed by seven blocks of 72 trials in which self-now, self-one, self-forty, stranger, and re-paired stimuli occurred equally often in a random order. In total, across all the blocks, there were 63 trials in each condition (i.e., self-now matching, self-now non-matching, self-one matching, self-one nonmatching, self-forty matching, self-forty nonmatching, stranger matching, stranger nonmatching). On completion of the task, participants were debriefed and dismissed.

## Results and discussion

### Perceptual matching

**Fig. 2:**
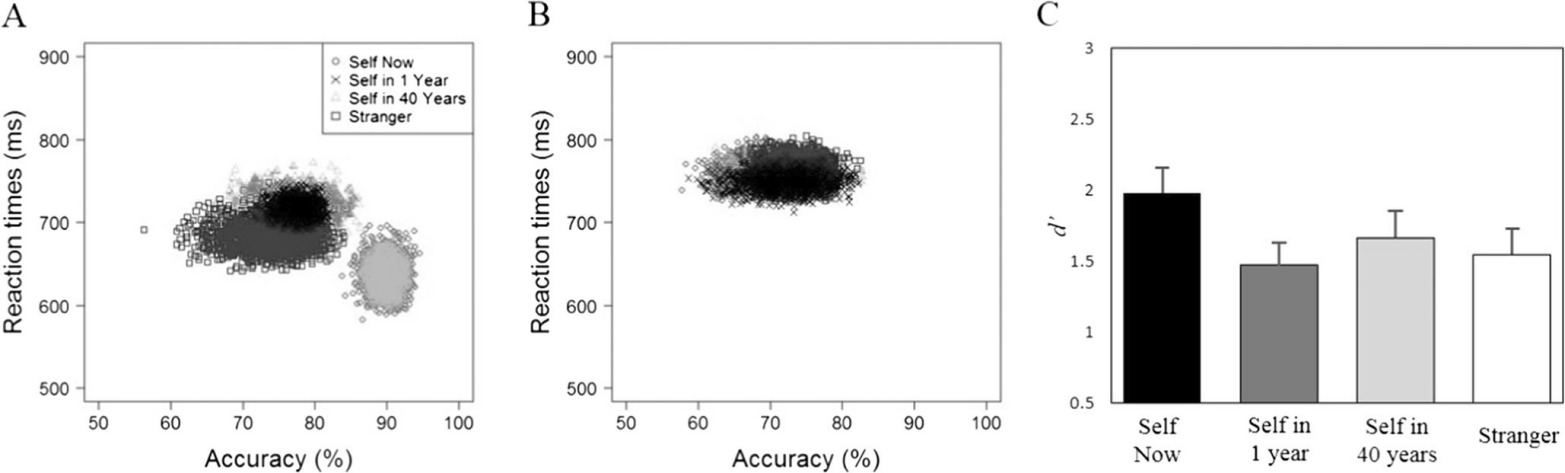
Distribution of bootstrapped sample means for matching (**a**) and nonmatching (**b**) trials (Experiment 1). The *x*-axis represents the accuracy rates and the *y*-axis the reaction times. **c** Shows *d'* as a function of shape category. *Error bars* represent +1 *SEM*

Responses faster than 200 ms were excluded from the analysis, eliminating less than 1% of the overall number of trials. Table 1 shows the accuracy and response time (RT) data. Following Sui et al. (2012), a bootstrapping procedure was adopted to examine the distribution characteristic of perceptual-matching judgments in each condition, combining accuracy and RT performance (Davison & Hinkley, 1997). For each participant in each condition, accuracy and RT were paired as a single data point (*x*, *y*). A bootstrapped data set was then created by re-sampling the data with replacement, keeping the sample size as the number of participants. The mean of this bootstrapped data set was then calculated and plotted as a single data point in the distribution (*x*, *y*). This procedure was repeated 2,000 times to estimate the population mean and variation for each condition. The resultant distributions across the different shape-category judgments are displayed in Fig. 2. Whereas the bootstrapped sample mean observations for self-now matching judgments fell in the lower right corner of the figure, all other matching judgments fell in an upper middle location (see Fig. 2a). In contrast, an overlapping distribution of observations emerged for responses to non-matching shape-category pairs (see Fig. 2b).

**Table 1:** Mean reaction times and accuracy as a function of shape category and trial type (Experiment 1)

Trial type	Shape category	Mean RT (ms)	Accuracy (%)
Matching	Self-now	637 (74)	90 (7)
	Self-one	718 (41)	77 (8)
	Self-forty	717 (65)	76 (15)
	Stranger	684 (65)	74 (16)
Nonmatching	Self-now	762 (53)	70 (14)
	Self-one	749 (44)	72 (16)
	Self-forty	756 (35)	73 (14)
	Stranger	764 (51)	73 (11)

*Note.* RT = reaction time. Standard deviations appear within parentheses

To explore the accuracy of participants’ responses, a signal detection approach was adopted. For each shape-category, performance in the matching and non-matching conditions were combined to calculate a measure of sensitivity (*d'*) and a single factor (shape category: self-now vs. self-one vs. self-forty vs. stranger) repeated-measures analysis of variance (ANOVA) was performed on these data (see Fig. 2c). This revealed an effect of shape category, *F*(3, 45) = 4.08, *p* = .012, η_p_^2^ = .21, such that *d'* was larger for self-now than for self-one, *t*(15) = 3.23, *p* = .003, *d* = .81, and stranger, *t*(15) = 2.22, *p* =.02, *d* = .55. The difference between self-now and self-forty was not significant, *t*(15) = 1.62, *p* = .063.

A 4 (shape category: self-now vs. self-one vs. self-forty vs. stranger) × 2 (trial type: matching or nonmatching) repeated-measures ANOVA on the RTs revealed main effects of shape category, *F*(3, 45) = 5.88, *p* = .002, η_p_^2^ = .28, trial type, *F*(1, 15) = 83.83, *p* < .001, η_p_^2^ = .85, and a significant Shape Category × Trial Type interaction, *F*(3, 45) = 15.68, *p* < .001, η_p_^2^ = .51. Further analyses yielded a significant simple effect of shape category on matching trials, *F*(3, 45) = 11.74, *p* < .001, η_p_^2^ = .44, such that RTs were faster for self-now than self-one, *t*(15) = -6.61, *p* < .001, *d* = 1.65, self-forty, *t*(15) = -3.67, *p* < .001, *d* = .92, and stranger, *t*(15) = -2.86, *p* = .006, *d* = .72. No other significant differences were observed.

Replicating Sui et al. (2012), these results demonstrate a self-prioritization effect on perceptual matching (Humphreys & Sui, 2015; Sui & Humphreys, 2015). Critically, however, performance was only enhanced when geometric shapes were associated with the current self. Future conceptions of the self produced patterns of performance equivalent to associating stimuli with a stranger, even when the self was construed only 1 year forwards in time (see Pronin et al., 2008; Pronin & Ross, 2006; Wakslak et al., 2008). This self-now prioritization effect confirms that temporal influences on self-construal impact perceptual matching.

### Diffusion modeling

To identify the processes underlying task performance during perceptual matching, data were submitted to a HDDM analysis. HDDM is an open-source software package written in Python for the hierarchical Bayesian estimation of drift diffusion model parameters (Wiecki et al., 2013). This approach assumes that the model parameters for individual participants are random samples drawn from group-level distributions and uses Bayesian statistical methods to estimate all parameters at both the group- and individual-participant level (Vandekerckhove, Tuerlinckx, & Lee, 2011). An advantage of this approach is that it is robust at recovering model parameters when less data (i.e., experimental trials) are available (Wiecki et al., 2013).

Models were response coded, such that the upper threshold corresponded to a matching response and the lower threshold to a nonmatching response. To test the hypothesis that self-prioritization is underpinned by a stimulus bias (Humphreys & Sui, 2015; Sui & Humphreys, 2015), separate drift rates (*v*) were estimated (i.e., allowed to vary) as a function of shape category and trial type (i.e., positive values = drift rate on matching trials, negative values = drift rate on nonmatching trials). A single starting value (*z*) was allowed to vary between the response thresholds, such that *z* = 0.5 indicates no bias (i.e., the starting point is located at the midpoint between the thresholds). Separate nondecisional processes (*t*_*0*_) were estimated for matching and non-matching trials (Voss, Rothermund, Gast, & Wentura, 2013). Bayesian posterior distributions were modeled using a Markov Chain Monte Carlo (MCMC) with 10,000 bootstraps (following 1,000 burn in samples). Prior to analysis, trials with latencies faster than 200 ms were removed, and the HDDM software removed the 5% of trials with the longest response latencies (Ratcliff & Tuerlinckx, 2002). In this model, bias during decisional processing could be mapped on to the drift rate (*v*), indicating a stimulus bias; or the position of the starting point (*z*), indicating a response bias (White & Poldrack, 2014).

**Fig. 3:**
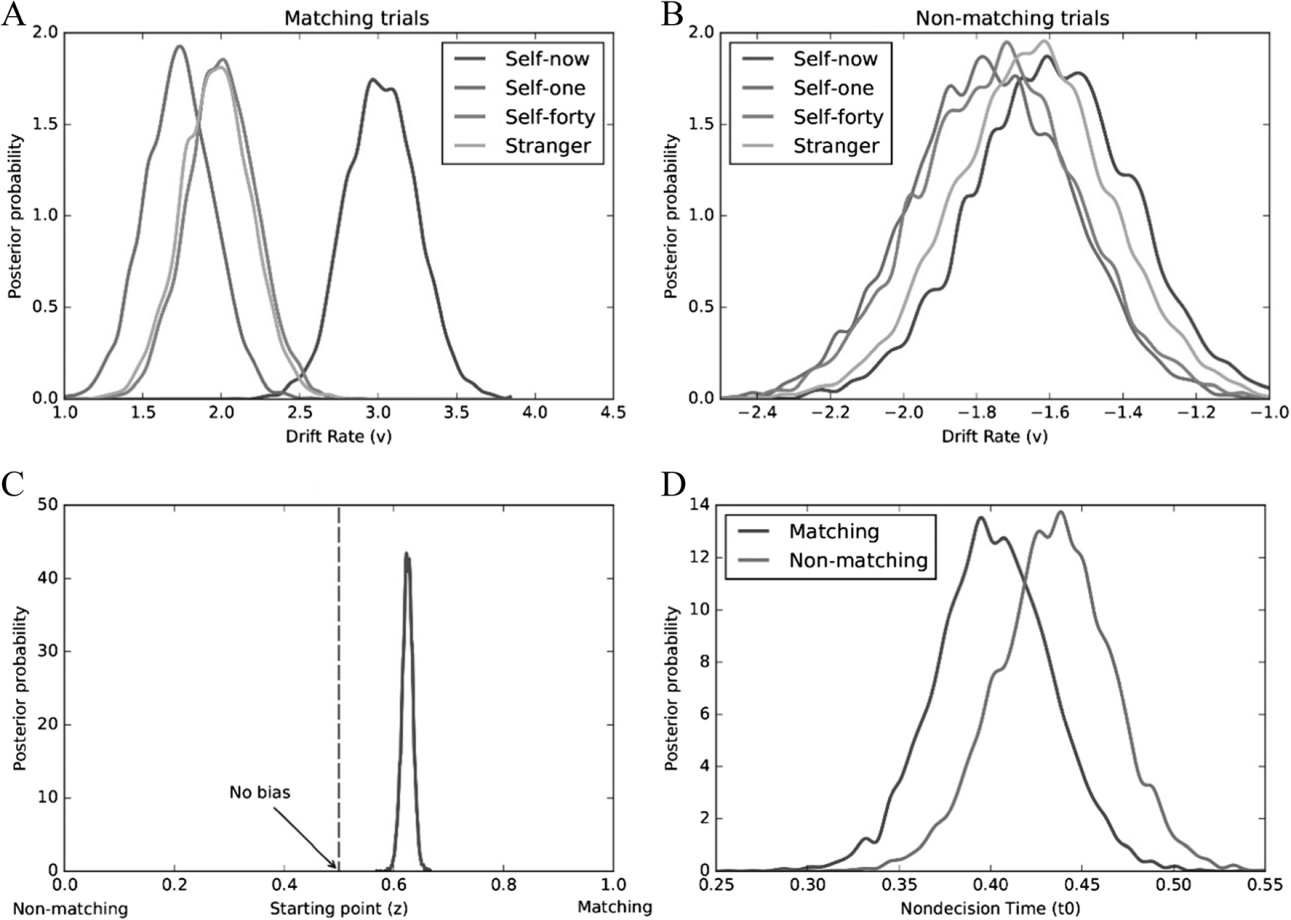
Mean posterior distributions of drift rate (*v*) as a function of shape-category and trial type (Expt. 1, **a—**matching trials; **b**—nonmatching trials). Mean posterior distribution of starting point (*z*) - (Expt. 1, **c**). Mean posterior distributions of nondecision time (*t*_*0*_) as a function of trial type (Expt. 1, **d**)

To determine the adequacy of this model, five additional models were tested for comparison. For the first two models, only the drift parameter (*v*) and starting point (*z*) were allowed to vary. For the other models, combinations of drift rate (*v*), starting point (*z*), and nondecision (*t*_*0*_) processes were allowed to vary. As can be seen in Table 2, the model that included all three parameters yielded the best fit (i.e., smallest DIC value). Interrogation of the posterior distributions revealed evidence of both stimulus and response biases during decision-making (see Fig. 3). Specifically, on matching trials, drift rates (*v*) were higher (i.e., evidence accumulation was faster) for self-now than all other shape-category pairings [*p*_Bayes_(self-now > self-one & self-forty & stranger) = 1.0]. This was not the case on nonmatching trials [*p*_Bayes_(self-now > self-one & self-forty & stranger) = .312]. In addition, comparison of the observed starting value (*z* = .62) with no bias (i.e., *z* = 0.5) revealed an a priori bias towards matching responses [*p*_Bayes_(bias > 0.5) = 1.0]. Finally, nondecisional processes (*t*_*0*_) yielded no difference between matching and non-matching trials [*p*_Bayes_(matching < nonmatching) = .777].

**Table 2:** Deviance information criterion (DIC) values for each model (Experiments 1–3)

DIC			
Models	Expt. 1	Expt. 2	Expt. 3
*v*	2141	1152	2859
*z*	5195	5054	5865
*v, z*	1429	463	2176
*z, t*_*0*_	4757	4674	5550
*v, t*_*0*_	1708	844	2726
*v, z, t*_*0*_	1125	83	1961

*Note. v* = drift rate, *z* = starting point, *t*_*0*_ = nondecision processes. A DIC difference of 2 is positive evidence for a model, greater than 10 is strong evidence for a model (Kass & Raftery, 1995).

These results explicate the basis of the self-prioritization effect on perceptual matching (Sui et al., 2012). Compared to other shape-category pairings (i.e., self-one, self-forty, stranger), self-now was characterized by a higher drift rate (*v*). This provides the first evidence that self-relevance enhances information uptake during perceptual matching (Humphreys & Sui, 2015; Sui & Humphreys, 2015). In addition, a bias in decisional processes relating to the position of the starting point (*z*) was also observed, indicating that participants favored matching over nonmatching responses.

## Experiment 2

n Experiment 1, we expected self-prioritization effects to emerge when geometric shapes were associated with one’s current (i.e., self-now) and near-future (i.e., self-one) self, but not with conceptions of the self in the distant future (i.e., self-forty). Interestingly, however, a different pattern of results was observed. Although stimulus prioritization was sensitive to temporal influences in self-construal (Pronin et al., 2008; Pronin & Ross, 2006; Trope & Liberman, 2003, 2010; Wakslak et al., 2008), only shapes associated with the current self yielded enhanced performance, a self-now prioritization effect that was underpinned by a stimulus bias during decisional processing (Sui et al., 2012). It is conceivable, however, that for undergraduates in their early 20s even a single year into the future feels temporally distant (Pronin et al., 2008), thereby eliminating prioritized processing. Acknowledging this possibility, our next study comprised a replication of Experiment 1 but with an important modification. On this occasion, together with current self and stranger, geometric shapes were associated with future self in a day and in a month. Of interest was whether narrowing the future horizon would impact the emergence of the self-prioritization effect. As in Experiment 1, to identify the processes underlying task performance, the data were submitted to a HDDM analysis (Wiecki et al., 2013)

## Participants and design

Sixteen undergraduates (three male, *M*_age_ = 20.88 years, *SD* = 2.45) took part in the research, for which they received £5 (~$6.20). All participants had normal or corrected-to-normal visual acuity. Informed consent was obtained from participants prior to the commencement of the experiment, and the protocol was reviewed and approved by the Ethics Committee at the School of Psychology, University of Aberdeen. The experiment had a 4 (shape category: self-now vs. self-day vs. self-month vs. stranger) × 2 (trial type: matching vs. nonmatching) repeated-measures design.

## Stimulus materials and procedure

Participants arrived at the laboratory individually, were greeted by an experimenter, and told they would be performing a perception task. The study closely followed Experiment 1 but with an important modification. On this occasion, during the learning phase, participants associated geometric shapes (i.e., circle, horizontal rectangle, cross, diamond) with self-now, self-in-a-day, self-in-a-month, and stranger. During the subsequent perceptual-matching task, participants had to report whether a series of shape-label pairings (e.g., circle & self-day, cross & self-month) were correct (or incorrect) on the basis of the associations learned previously. As in Experiment 1, participants initially performed 18 practice trials, followed by seven blocks of 72 trials in which self-now, self-day, self-month, stranger, and re-paired stimuli occurred equally often in a random order. In total, across all the blocks, there were 63 trials in each condition. On completion of the task, participants were debriefed and dismissed.

## Results and discussion

### Perceptual matching

**Fig. 4:**
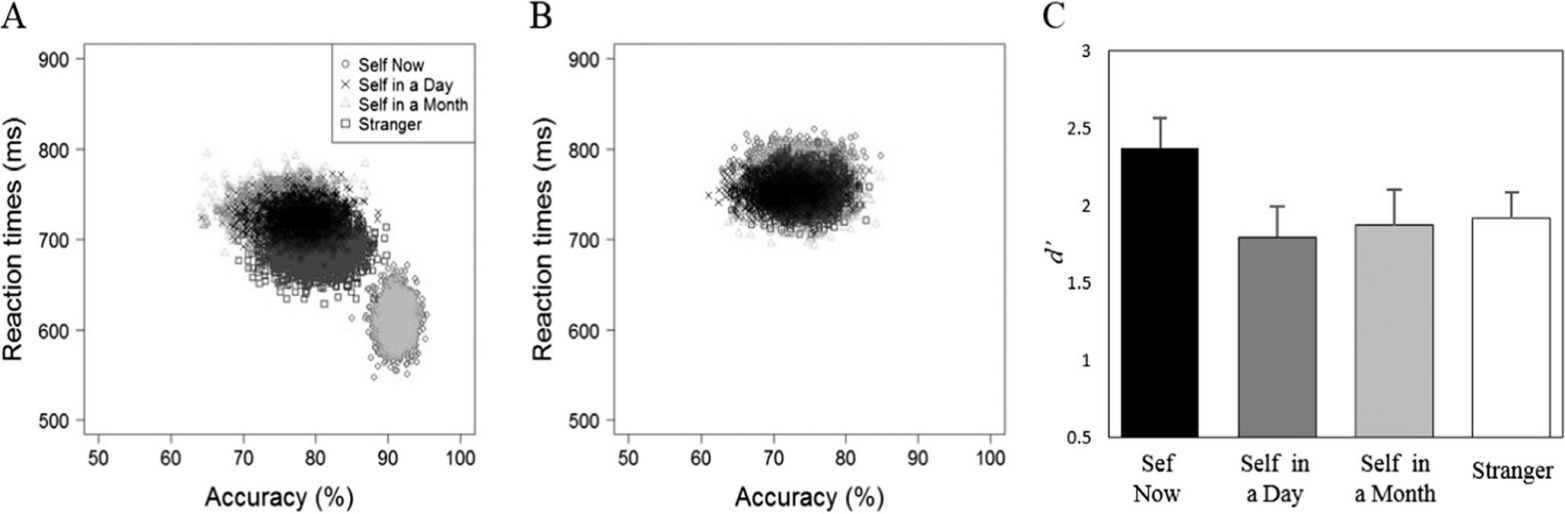
Distribution of bootstrapped sample means for matching (**a**) and nonmatching (**b**) trials (Expt. 2). The *x*-axis represents the accuracy rates and the *y*-axis the reaction times. **c** Shows *d'* as a function of shape category. *Error bars* represent +1 *SEM*

Responses faster than 200 ms were excluded from the analysis, eliminating less than 1% of the overall number of trials. Table 3 shows the accuracy and response time (RT) data. As in Experiment 1, a bootstrapping procedure was adopted to examine the distribution characteristic of perceptual-matching judgments in each condition, combining accuracy and RT performance. The resultant distributions across the different shape-category judgments are displayed in Fig. 4. Whereas the bootstrapped sample mean observations for self-now matching judgments fell in the lower right corner of the figure, all other matching judgments fell in an upper middle location (see Fig. 4a). In contrast, an overlapping distribution of observations emerged for responses to non-matching shape-category pairs (see Fig. 4b).

**Table 3:** Mean reaction times and accuracy as a function of shape category and trial type (Experiment 2)

Trial type	Shape category	Mean RT (ms)	Accuracy (%)
Matching	Self-now	611 (78)	91 (6)
	Self-day	724 (62)	77 (15)
	Self-month	730 (73)	77 (14)
	Stranger	688 (73)	79 (13)
Nonmatching	Self-now	778 (57)	73 (13)
	Self-day	753 (55)	72 (14)
	Self-month	746 (62)	74 (14)
	Stranger	753 (62)	74 (11)

*Note.* RT = reaction time. Standard deviations appear within parentheses

As before, a signal detection approach was adopted to explore the accuracy of participants’ responses. For each shape, performance in the matching and nonmatching conditions were combined to calculate a measure of sensitivity (*d'*) and submitted to a single factor (shape category: self-now vs. self-day vs. self-month vs. stranger) repeated-measures ANOVA (see Fig. 4c). This revealed an effect of shape category, *F*(3, 45) = 5.77, *p* = .002, η_p_^2^ = .28, such that *d'* was larger for self-now than self-day, *t*(15) = 4.55, *p* < .001, *d* = 1.14, self-month, *t*(15) = 3.33, *p* = .002, *d* = .83, and stranger, *t*(15) = 2.58, *p* =.01, *d* = .64.

A 4 (shape category: self-now vs. self-day vs. self-month vs. stranger) × 2 (trial type: matching or nonmatching) repeated-measures ANOVA on RTs revealed main effects of shape category, *F*(3, 45) = 10.21, *p* < .001, η_p_^2^ = .41, trial type, *F*(1, 15) = 108.08, *p* < .001, η_p_^2^ = .88, and a significant Shape Category × Trial Type interaction, *F*(3, 45) = 33.24, *p* < .001, η_p_^2^ = .69. Further analyses yielded a significant simple effect of shape category on matching trials, *F*(3, 45) = 25.28, *p* < .001, η_p_^2^ = .63, such that RTs were faster for self-now than self-day, *t*(15) = -9.23, *p* < .001, *d* = 2.31; self-month, *t*(15) = -6.38, *p* < .001, *d* = 1.60; and stranger, *t*(15) = -4.69, *p* < .001, *d* = 1.17. In addition, RTs were faster for stranger than self-month, *t*(15) = 2.71, *p* = .029, *d* = 0.92. A simple effect of shape category also emerged on nonmatching trials, *F*(3, 45) = 5.84, *p* = .005, η_p_^2^ = .28, indicating that RT’s were slower for self-now than self-day, *t*(15) = 2.84, *p* = .006, *d* = .71; self-month, *t*(15) = 2.96, *p* = .005, *d* = .74; and stranger, *t*(15) = 2.93, *p* = .005, *d* = .73. No other significant differences were observed.

Replicating Experiment 1, these results demonstrate a self-prioritization effect on perceptual matching (Humphreys & Sui, 2015; Sui & Humphreys, 2015). As previously, however, prioritized processing only emerged when geometric shapes were associated with the current self (i.e., self-now prioritization effect). Even for characterizations of the self in the very near future (i.e., tomorrow, in a month) self-relevance did not facilitate performance (i.e., self = stranger). This self-now prioritization effect corroborates the contention that temporal influences on self-construal influence perceptual matching.

## Diffusion modeling

**Fig. 5:**
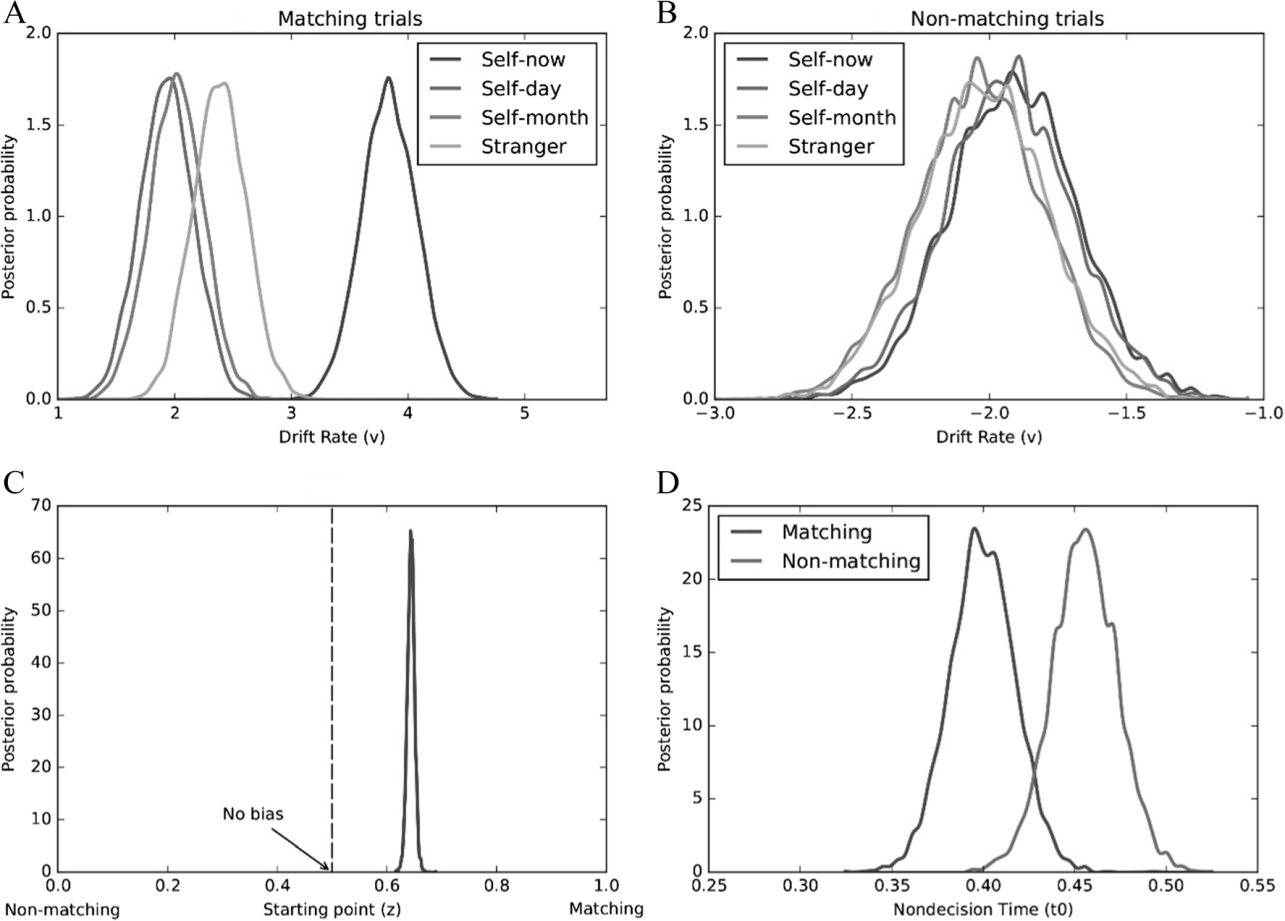
Mean posterior distributions of drift rate (*v*) as a function of shape-category and trial type (Expt. 2, **a**—matching trials; **b**—nonmatching trials). Mean posterior distribution of starting point (*z*) - (Expt. 2, **c**). Mean posterior distributions of nondecision time (*t*_*0*_) as a function of trial type (Expt. 2, **d**)

To identify the processes underlying task performance, data were submitted to a HDDM analysis (Wiecki et al., 2013). As in Experiment 1, five additional models were tested for comparison. As can be seen in Table 2, the model that included all three parameters yielded the best fit (i.e., smallest DIC value). Interrogation of the posterior distributions revealed evidence of both stimulus and response biases during perceptual matching (see Fig. 5). Specifically, on matching trials, drift rates (*v*) were higher (i.e., evidence accumulation was faster) for self-now than all other shape-category pairings [*p*_Bayes_(self-now > self-day & self-month & stranger) = 1.0]. This was not the case on nonmatching trials [*p*_Bayes_(self-now > self-day & self-month & stranger) = .376]. In addition, comparison of the observed starting value (*z* = .64) with no bias (i.e., *z* = 0.5) revealed an a priori bias towards matching responses [*p*_Bayes_(bias > 0.5) = 1.0]. Finally, nondecisional processes (*t*_*0*_) were faster on matching than nonmatching trials [*p*_Bayes_(matching < nonmatching) = .986].

These results directly replicate the pattern of effects reported in Experiment 1. Compared to all other shape-category pairings (i.e., self-day, self-month, stranger), self-now was characterized by a larger drift rate (*v*), thus supporting the contention that self-relevance enhances information uptake during perceptual matching (Humphreys & Sui, 2015; Sui & Humphreys, 2015). As previously, a bias in decisional processes relating to the position of the starting point (*z*) was also observed. Specifically, participants favored matching over nonmatching responses. Corroborating Experiment 1, these findings confirm that, at least in the context of a shape-label matching task, self-prioritization is underpinned by a bias in stimulus processing (Sui et al., 2012).

## Experiment 3

Thus far, the results have revealed a self-now prioritization effect, such that perceptual-matching is enhanced when one’s current self is contrasted with representations of the self in the distant (i.e., Expt. 1) and near (i.e., Expt. 2) future. What this demonstrates is that perceptual matching is influenced by temporally induced differences between one’s current and future (i.e., hypothetical) selves (Trope & Liberman, 2003, 2010). It is worth noting, however, that the effects of temporal construal also extend to representations of the self in the past, representations grounded in actual experience (e.g., Frank & Gilovich, 1989; Libby & Eibach, 2002; Nigro & Neisser, 1983; Pronin & Ross, 2006; Robinson & Swanson, 1993). Echoing the principles of CLT (Trope & Liberman, 2003, 2010)—despite detailed knowledge of one’s prior self (e.g., recollections of last week/month/year)—self-relevant cognitions and memories increase in abstraction as one travels backwards in time (e.g., Broemer, Grabowski, Gebauer, Ermel, & Diehl, 2008; Conway & Pleydell-Pearce, 2000; D’Argembeau & Van der Linden, 2004; Johnson, Foley, Suengas, & Raye, 1988). This then raises an interesting question. Is the self-prioritization effect similarly sensitive to representations of the self in the past? We explored this issue in our final experiment. As previously, a HDDM analysis was used to interrogate the cognitive processes underpinning task performance (Wiecki et al., 2013).

## Method

### Participants and design

Sixteen undergraduates (three male, *M*_age_ = 20.44 years, *SD* = 1.67) took part in the research, for which they received £5 (~$6.20). All participants had normal or corrected-to-normal visual acuity. Informed consent was obtained from participants prior to the commencement of the experiment and the protocol was reviewed and approved by the Ethics Committee at the School of Psychology, University of Aberdeen. The experiment had a 4 (shape category: self-now vs. self-day vs. self-month vs. stranger) × 2 (trial type: matching vs. nonmatching) repeated-measures design.

## Stimulus materials and procedure

Participants arrived at the laboratory individually, were greeted by a male experimenter, and told they would be performing a perception task. The study closely followed Experiment 2 but with an important modification. On this occasion, during the learning phase, participants associated geometric shapes (i.e., circle, horizontal rectangle, cross, diamond) with self-now, self-a-day-ago, self-a-month-ago, and stranger. During the subsequent perceptual-matching task, participants had to report whether a series of shape-label pairings (e.g., circle & self-day, cross & self-month) were correct (or incorrect) on the basis of the associations learned previously. As in Experiment 2, participants initially performed 18 practice trials, followed by seven blocks of 72 trials in which self-now, self-day, self-month, stranger, and re-paired stimuli occurred equally often in a random order. In total, across all the blocks, there were 63 trials in each condition. On completion of the task, participants were debriefed and dismissed.

## Results and discussion

### Perceptual matching

**Fig. 6:**
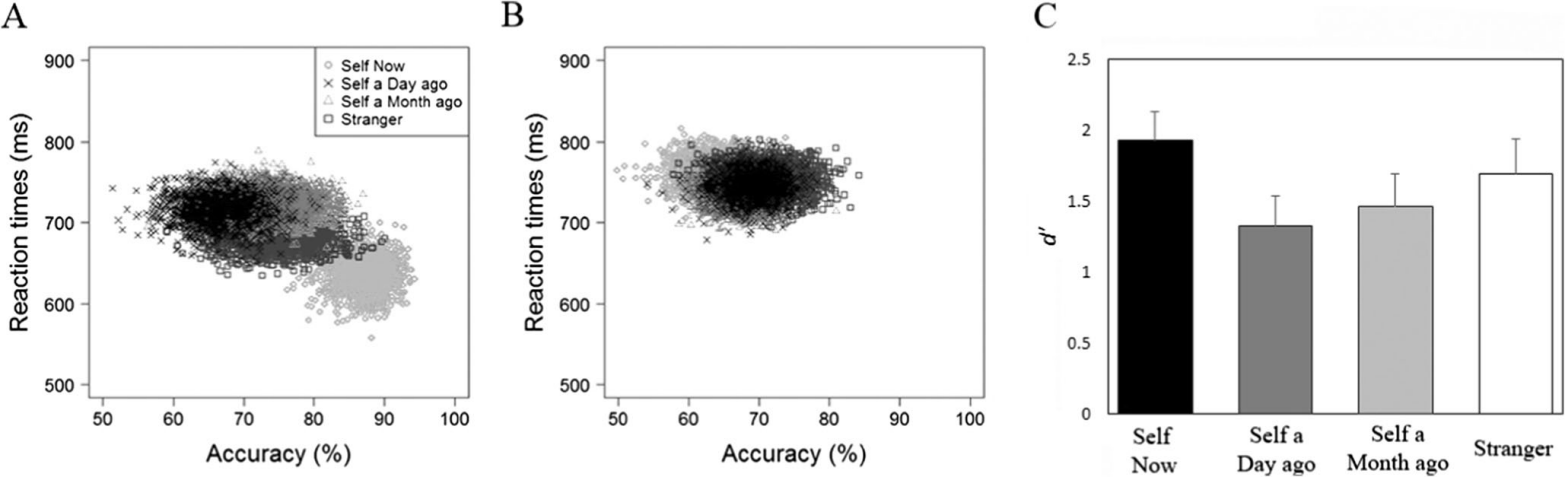
Distribution of bootstrapped sample means for matching (**a**) and nonmatching (**b**) trials (Expt. 3). The *x*-axis represents the accuracy rates and the *y*-axis the reaction times. **c** Shows *d'* as a function of shape category. *Error bars* represent +1 *SEM*

Responses faster than 200 ms were excluded from the analysis, eliminating less than 1% of the overall number of trials. One participant failed to follow the instructions, thus was excluded from the analysis. Table 4 shows the accuracy and response time (RT) data. As in Experiments 1 and 2, a bootstrapping procedure was adopted to examine the distribution characteristic of perceptual-matching judgments in each condition, combining accuracy and RT performance. The resultant distributions across the different shape-category judgments are displayed in Fig. 6. Whereas the bootstrapped sample mean observations for self-now matching judgments fell in the lower right corner of the figure, all other matching judgments fell in an upper middle location (see Fig. 6a). In contrast, an overlapping distribution of observations emerged for responses to nonmatching shape-category pairs (see Fig. 6b).

**Table 4:** Mean reaction times and accuracy as a function of shape category and trial type (Experiment 3)

Trial type	Shape category	Mean RT (ms)	Accuracy (%)
Matching	Self-now	628 (84)	87 (11)
	Self-day	715 (76)	65 (18)
	Self-month	723 (66)	72 (18)
	Stranger	684 (62)	73 (19)
Nonmatching	Self-now	761 (70)	63 (14)
	Self-day	745 (65)	67 (13)
	Self-month	739 (66)	66 (13)
	Stranger	752 (66)	70 (15)

*Note.* RT = reaction time. Standard deviations appear within parentheses

As previously, a signal detection approach was adopted to explore the accuracy of participants’ responses. For each shape, performance in the matching and non-matching conditions were combined to calculate a measure of sensitivity (*d'*) and submitted to a single factor (shape category: self-now vs. self-day vs. self-month vs. stranger) repeated-measures ANOVA (see Fig. 6c). This revealed an effect of shape category, *F*(3, 42) = 5.79, *p* = .002, η_p_^2^ = .29, such that *d'* was larger for self-now than for self-day, *t*(14) = 3.87, *p* = .001, *d* = 1.03; self-month, *t*(14) = 2.98, *p* = .012, *d* = .87; but not for stranger, *t*(14) = 1.50, *p* =.21.

A 4 (shape category: self-now vs. self-day vs. self-month vs. stranger) × 2 (trial type: matching or nonmatching) repeated-measures ANOVA on RTs revealed main effects of shape category, *F*(3, 42) = 6.95, *p* < .001, η_p_^2^ = .33; trial type, *F*(1, 14) = 86.48, *p* < .001, η_p_^2^ = .86; and a significant Shape Category × Trial Type interaction, *F*(3, 42) = 15.17, *p* < .001, η_p_^2^ = .52. Further analyses yielded a significant simple effect of shape category on matching trials, *F*(3, 42) = 14.22, *p* < .001, η_p_^2^ = .50, such that RTs were faster for self-now than self-day, *t*(14) = -5.38, *p* < .001, *d* = 1.60; self-month, *t*(14) = -5.88, *p* < .001, *d* = 1.21; and stranger, *t*(14) = -3.45, *p* = .003, *d* = .81. No other significant differences were observed.

Replicating Experiments 1 and 2, these results demonstrate a self-prioritization effect on perceptual matching (Humphreys & Sui, 2015; Sui & Humphreys, 2015). As previously, however, prioritized processing only emerged when geometric shapes were associated with the current self (i.e., self-now prioritization effect). Even for characterizations of the self in the immediate past (i.e., yesterday) self-relevance did not facilitate performance. Thus, extended to representations in the past (Trope & Liberman, 2003, 2010), this self-now prioritization effect corroborates the contention that temporal influences on self-construal influence perceptual matching.

## Diffusion modeling

**Fig. 7:**
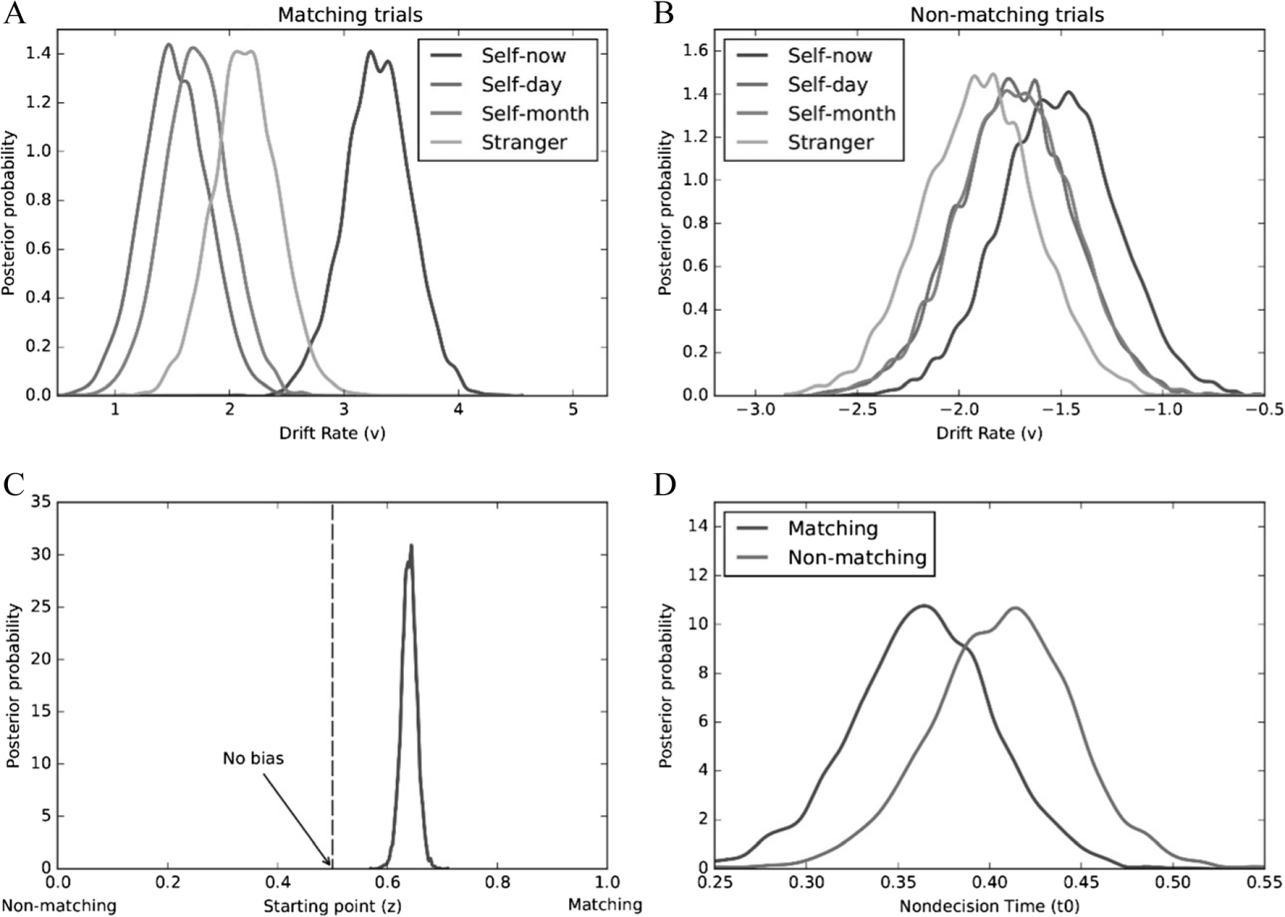
Mean posterior distributions of drift rate (*v*) as a function of shape-category and trial type (Expt. 3, **a**—matching trials; **b**—nonmatching trials). Mean posterior distribution of starting point (*z*) - (Expt. 3, **c**). Mean posterior distributions of nondecision time (*t*_*0*_) as a function of trial type (Expt. 3, **d**)

To identify the processes underlying task performance, data were submitted to a HDDM analysis (Wiecki et al., 2013). To determine the adequacy of this model, five additional models were tested for comparison. As can be seen in Table 2, the model that included all three parameters yielded the best fit (i.e., smallest DIC value). Interrogation of the posterior distributions revealed evidence of both stimulus and response biases during perceptual matching (see Fig. 7). Specifically, on matching trials, drift rates (*v*) were higher (i.e., evidence accumulation was faster) for self-now than all other shape-category pairings [*p*_Bayes_(self-now > self-day & self-month & stranger) = 1.0]. This was not the case on nonmatching trials [*p*_Bayes_(self-now > self-day & self-month & stranger) = .202]. In addition, comparison of the observed starting value (*z* = .62) with no bias (i.e., *z* = 0.5) revealed an a priori bias towards matching responses [*p*_Bayes_(bias > 0.5) = 1.0]. Finally, nondecisional processes (*t*_*0*_) yielded no difference between matching and non-matching trials [*p*_Bayes_(matching < non-matching) = .790].

Extended to representations of the self in the past, these results further explicate the basis of the self-prioritization effect on perceptual matching (Sui et al., 2012). Compared to all other shape-category pairings (i.e., self-day, self-month, stranger), self-now was characterized by a higher drift rate (*v*). This supports the contention that self-relevance enhances information uptake during perceptual matching (Humphreys & Sui, 2015; Sui & Humphreys, 2015). In addition, a bias in decisional processes relating to the position of the starting point (*z*) was also observed, indicating that participants favored matching over non-matching responses. Thus, replicating Experiments 1 and 2, the self-prioritization effect during perceptual matching was underpinned by a bias in stimulus processing.

## General discussion

A rapidly emerging literature has revealed a pervasive self-related bias in perceptual matching (Humphreys & Sui, 2015; Sui et al., 2012; Sui & Humphreys, 2015; Sui, Liu, et al., 2013; Sui et al., 2014). Specifically, association with the self (vs. friend and stranger) facilitates the processing of otherwise meaningless geometric shapes. Developing this line of inquiry, here we considered the extent and basis of this self-prioritization effect. Across three experiments, a consistent pattern of results was observed.^5^ First, prioritized processing was sensitive to temporal influences on self-construal (Pronin et al., 2008; Pronin & Ross, 2006; Trope & Liberman, 2003, 2010), such that self-prioritization only emerged when stimuli were associated with the current self (i.e., self-now prioritization effect). This effect, moreover, was insensitive to the direction of temporal construal. A prioritization effect was observed when current self was compared to representations of the self in both the future (i.e., Expts. 1 & 2) and past (i.e., Expt. 3). Second, a HDDM analysis revealed that task performance was underpinned by both stimulus and response biases. While participants favored matching over nonmatching responses in all three experiments—more critically, self-prioritization was underpinned by differences in the rate of information uptake (*v*). Specifically, self-now was characterized by a higher drift rate than all other shape-category pairings. These findings are noteworthy in the current academic climate as they demonstrate the replicability of the self-prioritization effect during perceptual matching across different temporal directions and timescales.

In showing that temporal influences on self-construal moderate the emergence of the self-prioritization effect (Humphreys & Sui, 2015; Sui & Humphreys 2015), the current work resonates closely with almost 2 decades of research exploring the effects of psychological construal on social-cognitive functioning (Trope & Liberman, 2003, 2010). The gist of construal-level theory is quite straightforward. Scaled egocentrically, psychological distance is anchored in one’s experience of the self in the here and now. What this means is that as objects and events—including the self—increase in distance (temporal & spatial) from this reference point, representations decrease in perceptual detail, specificity, and self-relevance. Indeed, on occasion, future selves are characterized and treated like other people (Hershfield, 2011; Mitchell et al., 2011; Parfit, 1971, 1987; Pronin et al., 2008; Pronin & Ross, 2006; Wakslak et al., 2008). At least in the context of perceptual matching, this self-becomes-other effect was observed only a single day into the future (and past), thereby revealing the primacy of the current self during sensorimotor processing (Conway, 2005).

That the self-prioritization effect was restricted to geometrical shapes associated with the current self underscores the nuanced character of self-referential processing (McConnell, 2011). A basic function of the self is to guide people’s interactions with the world (Higgins, 1996). This can best be achieved not through the deployment of a rigid, unitary entity (Humphreys & Sui, 2015; Sui & Humphreys, 2015), but rather through the application of context-dependent representations (i.e., multiple selves) that are sensitive to the demands of the immediate situation (Ainslie, 1992; Kurzban & Aktipis, 2007; H. R. Markus & Nurius, 1986; McConnell, 2011; Parfit, 1971; Schelling, 1984; Thaler & Shefrin, 1981). From this perspective, it is unsurprising that temporal factors influence stimulus prioritization. By necessity, the version of the self that is called upon in immediate task contexts serves a quite distinct function to the one that simulates future events and experiences (Gilbert & Wilson, 2007, 2009; Smallwood & Schooler, 2006). In particular, in the here and now, greater emphasis is placed on the construction of perceptually rich event (and self) representations (Trope & Liberman, 2003, 2010). Echoing this viewpoint, Conway and Pleydell-Pearce (2000) characterize the current self as a complex set of active goals and associated self-constructs in working memory that guide cognition flexibility and adaptively from one moment to the next (see also Conway, 2005). As demonstrated herein, the outputs of the current self also extend to perceptual processing. Consistent with the tenets of construal-level theory (Trope & Liberman, 2003, 2010), the self-prioritization effect in perceptual matching was confined to stimuli associated with the current self.

In the available research to date, the self-prioritization effect is considered to be a perceptual phenomenon. Noting how perception can seemingly be modified by characteristics of the observer—including desires, values, and expectancies (e.g., Collins & Olson, 2014; Dunning & Balcetis, 2013; Lupyan, 2015; Vetter & Newen, 2014)—self-relevance is believed to exert a comparable influence on stimulus processing (Humphreys & Sui, 2015; Sui et al., 2012; Sui & Humphreys, 2015). A primary purpose of the current investigation was therefore to explore the processes underpinning task performance during perceptual matching using a HDDM approach (Wiecki et al., 2013). This confirmed that decisional evidence was accumulated most rapidly when geometric shapes were associated with self-now than all other shape-label pairings. What this reveals is that, at least in the context of perceptual matching, self-relevance influences stimulus processing (i.e., the rate of information uptake) during decision making (Humphreys & Sui, 2015; Sui & Humphreys, 2015). What remains to be seen, however, is whether self-relevance operates in a comparable way in other judgmental settings (e.g., Macrae, Visokomogilski, Golubickis, Cunningham, & Sahraie, 2017). Future research should explore this issue.

Here, we have shown when and how temporal influences on self-construal moderate the emergence of the self-prioritization effect during a shape-label matching task (Sui et al., 2012). Of course, the effects of self-relevance likely extend well beyond such a laboratory-based activity. Take, for example, object classification. Continual interaction with a complex and demanding environment necessitate that a fundamental function of the self is to classify objects based on their personal significance and meaning (e.g., mine vs. not mine, goal-relevant vs. goal-irrelevant; Berlad & Pratt, 1995; Constable, Kritilos, & Bayliss, 2011; Constable, Kritikos, Lipp, & Bayliss, 2014; Fischler, Jin, Boaz, Perry, & Childers, 1987; Folmer & Yingling, 1997; Gray, Ambady, Lowenthal, & Deldin, 2004; Miyakoshi, Nomura, & Ohira, 2007; Müller & Kutas, 1996). One’s possessions are an obvious case in point, as evidenced by the fact that even young children understand the concept of ownership and afford it social significance (Fasig, 2000; Furby, 1980; Hay, 2006). When the perils of misappropriating other people’s effects (e.g., pint of beer) can be substantial (e.g., an indecorous altercation in the pub), the ability to discriminate items that one owns from items that one does not is an invaluable skill (Constable et al., 2011; Constable et al., 2014). As such, one might expect ownership to enhance object classification.

In considering the wider implications of the current findings, other core facets of self-construal likely play a prominent role in determining the products of self-referential processing. As noted, each person’s self comprises a collection of multiple, context-dependent identities (e.g., humanitarian, golfer, curry lover) that vary in both centrality and significance (Higgins, 1987; H. R. Markus & Nurius, 1986; McConnell, 2011; Roberts & Donahue, 1994). Acknowledging this representational complexity, theories of identity-based motivation contend that when a specific component of the self is activated (i.e., a person’s working self), processing resources are preferentially allocated to stimuli that bolster and enhance that identity if it is important to the individual (Oyserman, 2007, 2009). Thus, attention is directed toward identity-consistent stimuli, while inconsistent information is neglected or downplayed (Berger & Heath, 2007; Coleman & Williams, 2015). The utility of this account lies in the flexibility it affords perceptual processing in complex settings. If stimulus relevance is identity-dependent, then a specific object (e.g., cheesecake) has the potential to modulate perceptual processing depending on which of an individual’s multiple identities has been activated (e.g., dieter vs. dessert lover). A useful task for future research will therefore be to investigate the effects of identity activation and identity strength on the associations forged between the self and meaningful objects (cf. geometric shapes) in consequential task contexts. In addition, extending the age range of participants to include older adults may be revealing, especially for retrospective representations of the self that may be shaped by identity-relevant knowledge.

Beyond shape-label associations, recent work has demonstrated that the self-prioritization effect in perceptual matching extends to action planning. Modifying Sui et al.’s (2012) paradigm, Frings and Wentura (2014) initially required participants to associate labels (i.e., self, mother, stranger, no label) with arbitrarily assigned movements (i.e., up, down, left, right). During the subsequent testing phase, following execution of a cued movement, one of the four labels appeared and participants had to indicate whether the action and label matched their earlier learning experience. Extending Sui et al. (2012), a self-prioritization effect emerged such that performance was better for self-relevant actions than all other label-action pairings. As in the current investigation, it is possible that temporal influences on event construal may moderate the elicitation of this self-action prioritization effect (Frings & Wentura, 2014). Supported by an extensive literature, action planning is shaped by the timing of an event (Fujita, Henderson, Eng, Trope, & Liberman, 2006; Liberman & Trope, 1998; Liviatan, Trope, & Liberman, 2008; Trope & Liberman, 2003, 2010; Vallacher & Wegner, 1989; Wakslak, Trope, Liberman, & Alony, 2006). Consider, for example, the act of traveling into town to purchase an expensive single malt whisky. If the event is about to happen in the here and now, low-level construal will generate details of how one’s objective can best be accomplished (e.g., getting into the car, parking at the liquor store, locating malts from Speyside). In contrast, the same activity in the future will tend to be characterized in a decontextualized way (i.e., high-level construal) that emphasizes only the overall meaning of the episode (e.g., purchasing a birthday gift). By implication, one might expect these temporal differences in the specificity of action planning to influence the emergence of self-prioritization effects (Frings & Wentura, 2014)

## Conclusion

In sum, here we demonstrated a critical determinant of the self-prioritization effect during perceptual matching (Sui et al., 2012) and identified the cognitive process through which this phenomenon arises. Specifically, stimulus prioritization was sensitive to temporal influences in self-construal (Trope & Liberman, 2003, 2010), such that only the association of geometric shapes with one’s current (vs. future/past) self facilitated task performance. In addition, drift diffusion modeling revealed that self-prioritization was underpinned by a stimulus bias (White & Poldrack, 2014). What has yet to be established, however, is whether comparable effects emerge in other task contexts in which stimuli vary in self-relevance.

## Footnotes

1 For additional information about diffusion modeling, see Ratcliff and McKoon’s website (The Diffusion Decision Model for Non-Specialists, http://star.psy.ohio-state.edu/wp/?page_id=169).

2 In each experiment, all multiple comparisons were corrected using the Holm-Bonferroni method (Holm, 1979).

3 Based on the average effect size reported by Sui et al. (2012), G*Power (*f* = .40, α = .05, power = 80%) revealed a requirement of 16 participants. This sample size was adopted for each of the reported experiments.

4 This manipulation of mental imagery has been shown to influence the specificity of self representation, triggering a switch from concrete to abstract self-construal as a function of increasing temporal distance (Macrae et al., 2015).

5 It has been suggested that females report more vivid imaginary experiences than males (Richardson, 1991). As participants in the current research used imagery to associate shapes with the self at different points in time, it is possible that the effects observed were amplified by the preponderance of females in each experiment. Future research should consider this possibility.
